# Supplementary material for: Preferences of healthcare workers using tongue swabs for tuberculosis diagnosis during COVID-19
Source: PLOS Glob Public Health. 2023 Sep 7;3(9):e0001430. doi: 10.1371/journal.pgph.0001430 (PMC10484421; doi:10.1371/journal.pgph.0001430)
Supplement: S2 Text — (DOCX) [file pgph.0001430.s002.docx]

## **S2 Text: SOP for supervised self-swabbing (SSS)**

*Note: All sample collection outside the clinical setting needs to be conducted in pairs and during the daytime while abiding by personnel security protocols of the partnering agency.

Swab Collection

1. The swabs are single-use, sterile, and packaged individually.
2. The healthcare worker (HCW) and patient should wear appropriate PPE.
3. Conditions for ideal sample collection (will vary for Aim 1):
   1. The patient should not eat, drink, brush their teeth or use mouthwash for at least 30 minutes before giving the sample.
   2. The sample should be collected in the morning, if possible.

*Copan FLOQ tongue swab: Supervised self-Swabbing*

1. Prior to swabbing, explain proper tongue swab collection to the patient.
2. Open the packaging and have the patient pull the swab out by the shaft. They should not touch the swab head.
3. The tongue swab will be collected by the patient following these directions:
   - 1. Firmly swab the length and breadth of the tongue dorsum, from the front to as far back as is comfortable.
     2. Press hard enough to slightly bend the shaft of the swab.
     3. Rotate the swab head during sampling.
     4. Swab for 15 seconds.
     5. Swabbing may leave the tongue feeling dry but should not cause any pain or discomfort.
   1. After sampling, the patient should put their mask back on with one hand and place the swab into the 2 mL tube held by the study staff member.
   2. The HCW will break off the head of the swab into the tube.
   3. Discard the shaft of the swab.
