# Supplementary material for: Preferences of healthcare workers using tongue swabs for tuberculosis diagnosis during COVID-19
Source: PLOS Glob Public Health. 2023 Sep 7;3(9):e0001430. doi: 10.1371/journal.pgph.0001430 (PMC10484421; doi:10.1371/journal.pgph.0001430)
Supplement: S3 Text — (DOCX) [file pgph.0001430.s003.docx]

**S3 Text: Interview Guide**

**User Acceptance and Risk Perceptions of Healthcare Workers Using Tongue Swabs for TB Diagnosis**

Thank you so much for taking time out of your day to hop on this call.

Hello my name is xxxx. I am a graduate student at the xxxx of Public Health. I work in the xxxx lab developing diagnostics from TB and COVID-19.

I work on improving diagnostic technologies and educational materials protecting workers in the healthcare setting. I will review the research statement and study procedures now and then we can have a discussion about your experiences.

**Researchers’ Statement**

**Welcome and Study Procedures**

Thank you for agreeing to participate in our study. We’ve asked you to participate because you are experienced in collecting samples for TB diagnostics using both the traditional sputum and tongue swab method. As participants in tongue swab sample collection activities in endemic settings during the era of COVID-19 we are interested in understanding what risks you are being exposed to and if you have the tools to mitigate these risks in your work. Each interview will last 45-60 minutes.

The purpose of this interview is to describe you and your experiences during sample collection from patients that are suspected to have TB during the era of COVID-19. We’d also like to hear your thoughts on potential recommendations that could minimize any threats and risks if any during future tongue swab sampling, including training and resource provision.

**Benefits and Risks to the Study**

There are no known personal benefits to participating in this study. There are minimal risks to your participation. It may benefit society and your occupation by providing more knowledge about perceived risks of using tongue swab in clinical practice. This understanding could lead to improving training and education materials for tongue swab and to inform protocols and procedures for tongue swab use.

**Explanation of the process**

We are using an interview format to help understand the context behind the answers you will provide to our questions, as we believe they will provide a more in-depth understanding than a written survey can provide. There are no wrong answers.

We will be taking notes and recording the interviews. Interviews will be professionally transcribed for data analysis. Interview data that has been de-identified may be placed in a data repository to answer alternative research questions. Your name or contact information will not be associated with any data that is shared in such a repository or with other researchers.

The findings may be written into a report or manuscript for publication in a peer-reviewed journal. We will not refer to you by name in any report or publication without your explicit written consent.

Your participation in this interview is completely voluntary and you may opt to stop at any point in the interview process and leave the study at any time. You will not be penalized for not answering questions or leaving the study. We will provide you with a complete copy of our results at the conclusion of the study.

In the next few weeks, I will send you a summary of the key points gleaned from your interview. I will ask that you review them and let me know if I accurately captured the perspectives you shared within two weeks of receiving that email.

**Questions and consent**

Do you have any questions before we begin?

Do you consent to be a participant in our study? (Ask participants to provide a verbal “yes”.)

Do you consent to the recording of this interview?

**Discussion** (threat susceptibility, threat severity, self-efficacy, response-efficacy)

**Intro**

We would like to hear about your background and experience with collecting samples for diagnosis.

1. Before we continue, how would you like me to refer to you as? Can you please pronounce your name?
2. What is your job title?
3. Can you tell me a little bit about your experience collecting samples for TB, including through sputum samples and tongue swab?
4. How long have you been working on this?
5. What language/es do you use in your work?
6. How would you describe your patient community?
7. How would you describe your community where you live?
8. How do you think your risk of getting TB at work compares to getting TB in your community?
9. How does your risk of getting TB compare to other risks of getting other diseases at work (e.g., COVID-19)?
10. What would be the consequences of getting TB at work?
    1. What are the consequences to you?
    2. What about your family?

**Background**

**Now, we’d like to hear a little bit more about how the risks that traditional sputum sample collection pose to your health and safety.**

1. How does collecting sputum to assess TB impact your risk of getting TB?
2. Do you feel like you have the knowledge, skills and ability to safely collect sputum samples?
   1. Posed: How do you reduce your risk of TB while taking samples?
3. Under what circumstances do you prefer to use the traditional sputum sampling collection method for TB diagnostics? Why?
   1. Prompt: Can you provide an example of a situation where you would prefer to use the traditional sputum sample as opposed to the oral swab?
4. Do you think your prior experience with sputum samples has helped you to mitigate risks of disease exposure? How? Why?
   1. To TB?
   2. To COVID-19? Other diseases?
      1. Prompt example: Because of a patient coughing while taking the sample, e.g., TB patient sick in hospital bed and not able to get outside to self-collect a sputum sample.

**Now, we’d like to hear a little bit more about how the risks that tongue swab poses to your health and safety.**

1. How does collecting samples to assess TB via tongue swab impact your risk of getting TB?
2. Do you feel like you have the knowledge, skills and ability to safely collect samples via tongue swab?
   1. Posed: How do you reduce your risk of TB while taking samples?
3. Under what circumstances do you prefer to use the tongue swab method for TB diagnostics? Why?
   1. Prompt: Can you provide an example of a situation where you would prefer to use tongue swab?
4. Do you think your prior experience with tongue swab has helped you mitigate risks of disease exposure? How? Why?
   1. To TB?
   2. To COVID-19? Other diseases?
      1. Prompt example: Because of a patient coughing while taking the sample, e.g., TB patient sick in hospital bed and not able to get outside to self-collect a sputum sample.

**Now, we’d like to hear a little bit more about how the risks that supervised patient self-swabbing poses to your health and safety. Reference link to supervised self-swab for flu test which I will extrapolate on for tongue swab for TB.**

1. How does collecting samples to assess TB via supervised patient self-swabbing impact your risk of getting TB?
2. Do you feel like you have the knowledge, skills and ability to implement self-swabbing?
   1. Prompt: Do you feel like your patients can safely and effectively collect their self-swab samples?
   2. Prompt: How do you reduce your risk of TB while taking samples?
3. How do you think collecting samples via patient self-swabbing affects your risk of getting TB?
4. Under what circumstances do you prefer to use the supervised tongue swab method for TB diagnostics? Why?
   1. Prompt: Can you provide an example of a situation where you would prefer to use tongue swab?
5. Do you think your prior experience with supervised self-swabbing has helped you mitigate risks of disease exposure? How? Why?
   1. To TB?
   2. To COVID-19? Other diseases?
      1. Prompt example: Because of a patient coughing while taking the sample, e.g., TB patient sick in hospital bed and not able to get outside to self-collect a self-swabbing sample.

**Prompt for flow of conversation:**

I know that you mentioned this….

Do you have any additional comments…

**Training**

1. Can you describe any training that you’ve received to protect yourself from TB exposure at work?
   - Prompt: Was any of this training specific to traditional sputum samples? To tongue swab? To self-swabbing?
2. Do you feel like your workplace conditions allow you to implement the training you received to protect your health and safety?
   - 1. What barriers exist in your workplace?
3. What additional training do you think you need to do your job safely?
4. What additional resources do you think you need to do your job safely?
5. How do you think your use of any of these methods influences the health and safety of other workers at your facility?
6. What training might they need to protect their health and safety if any?

**Closing**

- Thank you so much for sharing your expertise and experiences. Is there anything else you would like to share before we go?
- Would you mind if we contact you if we have additional questions in the future?
- Thank you very much for your participation. Wishing you and your team well during these uncertain times.
