# Supplementary material for: Preferences of healthcare workers using tongue swabs for tuberculosis diagnosis during COVID-19
Source: PLOS Glob Public Health. 2023 Sep 7;3(9):e0001430. doi: 10.1371/journal.pgph.0001430 (PMC10484421; doi:10.1371/journal.pgph.0001430)
Supplement: S1 Table — (DOCX) [file pgph.0001430.s004.docx]

## **S1 Table: Codebook and definitions**

| **Code** | **Short- hand** | **Examples of when to apply** | **Description** |
| --- | --- | --- | --- |
| **Attributes** | | | |
| **Participants’ Demographics** | Demog | "Years in the field" or "makes home visits." | Apply to key background information about the key informant relevant to the research, e.g., professional or personal demographics to help keep the inductive codes linked to the individual participants |
| **Interesting fact** | Fun Fact | How COVID-19 is impacting precautionary behaviors for TB. | Apply to key points that are interesting facts that may guide creation of codes for the inductive process. |
| **Great quote** | Quote | For phrases or sentences that might be particularly great to include in a final document  “I think sputum is gross and messy and I like tongue swab because it is cleaner and easier to use.”  ie) comparing the two methods Sputum vs tongue swab. Any context specific reasoning supporting their sample collection method of preference. Any ideas on improving safety. | Apply to particularly great or well-phrased quotes from the interview which can be used when reporting findings. |
| **Provider Swab** | HCW Swab | I prefer to take the samples myself because I don’t think the patients can do it right. | Apply to discussion about provider swabbing co-coded with relevant parent/child codes. |
| **Supervised Self Swab** | SS Swab | I don’t want to be so close to the patient with their mouth open so I prefer to have them do it themselves and I can guide them from a safe distance. | Apply to discussion about supervised self-swabbing co-coded with relevant parent/child codes. |
| **Comparison of Tongue Swab with Gold Standard Sputum Sampling** | tongue swab vs gold | Sputum is hard for many of my patients to produce so tongue swab would help ensure that those patients can still get a TB diagnosis test. | Apply to discussion comparing tongue swab to traditional sputum sampling which is the “Gold Standard” for TB diagnostics co-coded with relevant parent/child codes. |
| **Patient Communities** | Patient Com | “The community is very open and eager to join our studies.” or “Now with COVID, community members are not as eager to join our studies because they think we are giving them a vaccine that they are afraid of.” | Apply to text that discusses key informants’ beliefs or perceptions about the patient community’s beliefs, perceptions or actions that may be related to facilitators or barriers to use or willingness to use tongue swab. |
| **Parent Codes** | | | |
| **Exposure Pathway** | ExpPathway | Being exposed to a patient with TB not using proper PPE | Apply to text that discusses the ways in which the key informant may be exposed to hazards that affect their safety or health in their workplace or in their community. |
| **Perceived Threat** | Threat | If I come into contact with TB there is a likelihood that I will have a severe outcome. | Apply to text that describes the likelihood that a threat (TB or COVID) will affect the key informant and/or how bad it will be if it does. |
| **Training** | Training | SOP’s and training to protect myself from exposure to TB while using the tongue swabs. | Apply to text that discusses skills or knowledge acquired through formal training prior to collecting samples using the tongue swab method. Formal training may include, but is not limited to, field training, live simulation, power points, reference guides and/or illustrations. |
| **Self-Efficacy** | S-Efficacy | I cannot control the patients who are intoxicated and cannot follow directions when collecting samples. The HCW does not find the SOP to be achievable (low self- efficacy).  The HCW is able to prevent getting exposed to TB by following the SOP. (e.g., HCW is able to effectively and easily prevent occupational acquired tuberculosis by using tongue swabs).  the HCW does not find the SOP to be achievable. | Apply to text that discusses perceptions related to the Skills and Knowledge necessary to implement the relevant diagnostic effectively to diagnose TB, including the perception the key informant has of being competent to perform associated tasks to control the risk of getting (TB/COVID/other). |
| **Response Efficacy** | R-Efficacy | I think that the tongue swab will be a great way to test patients who are challenged with producing sputum for the “gold standard” TB diagnostics. | Apply to text that describes perceptions of effectiveness of relevant diagnosis test (PS, SSS, Gold Standard) for **detecting TB**. Can I successfully collect a sample for diagnosis? |
| **Mitigation Strategies** | Mitigation | I have all the PPE I need when collecting tongue swab samples. | Apply to text that describes strategies that were used to control/ mitigate occupational health hazards from spreading at work or in the community. (TB/COVID/other) |
| **Challenges** | Challenge | Going into the home early in the morning to collect samples and all the family is in the house. Kids jump on my lap and it is hard to maintain a safe physical distance. | Apply to text that describes challenges faced in the workplace that are perceived to impact the ability to implement tongue swab or Gold Standard safely or effectively. |
| **Opportunities** | Opportunity | training videos or an illustration to use to help explain how the patient can swab themselves while being supervised. | Apply to text that describes resources or systems that the key informant does not currently have access to but perceive would facilitate the safe or effective tongue swab sample collection process. |
| **Pre COVID-19** | Pre-COVID | “Before COVID we did not wear masks with the babies but now with COVID we wear masks all the time”. | Apply to text that describes behaviors, beliefs, or perceptions before COVID vs during the COVID pandemic. |
| **Social Support** | Support | I have social support from my colleagues when one of them volunteers to accompany me to make home visits. | Discusses how team dynamics and/or support systems can be used to reduce occupational health risk while collecting tongue swab samples. |
| **Child Codes** | | | |
| **Exposure Pathway/Workplace Exposure Pathway** | Work Exp | When a patient coughs in your face while collecting the sample | Apply to text that discusses the ways in which the key informant may be exposed to hazards that affect their safety or health in their workplace. |
| **Exposure Pathway/Community Exposure Pathway** | Community Exp | When a neighbor has active TB | Apply to text that discusses the ways in which the key informant may be exposed to hazards that affect their safety or health in their community. |
| **Training/ Provider Training** | Training Provider | I received an SOP on how to safely using the PS use the tongue swab | Apply to text that describes types of provider training, and/or issues related to provider training, on how to use the diagnostic (PS, SSS, or gold standard) |
| **Training/ Patient Training** | Training Patient | I received training on how to demonstrate to my patients how to safely swab themselves while I am watching them. | Apply to a text that describes types of patient training, and/or issues related to patient training, on how to use diagnostic (PS, SSS, or gold standard). |
| **Perceived Threat /Susceptibility COVID-19** | COVID Susceptibility | I got COVID-19 when we were in lockdown and I was not going to work so I know it was not occupationally acquired. If I can get TB in my community, I can also get it at work if I am not careful. Or: They don't think that they could get COVID-19 by using the traditional sputum sampling method and would rather not change to tongue swabs. | Discusses perceptions of the likelihood that a threat (COVID) will affect the key informant and/or cause them harm. |
| **Perceived Threat/ Susceptibility TB** | TB Susceptibility | I have been working with TB for 15 years now and have not been infected. I eat healthy and take care of myself so I won’t get TB. | Discusses perceptions of the likelihood that a threat (TB) will affect the key informant and/or cause them harm. |
| **Perceived Threat/ Severity COVID-19** | COVID Consequences | There isn’t a cure or treatment for COVID-19 so I am concerned with getting COVID-19 and maybe being seriously ill. | Describes the perception of the severity of consequences that exposure to a threat (COVID-19) will result. |
| **Perceived Threat/ Severity TB** | TB Consequences | There is a cure and treatment for TB so if I get it at work that is a part of the risk, I accept to do my job. I can easily do the treatment and I will be OK. | Describes the perception of the severity of consequences that exposure to a threat (TB) will result. |
| **PS Swab/**  **Willingness to use Provider Swabbing** | Use HCW | I don’t trust my patient who is intoxicated to swab themselves so I would prefer to swab them myself. | Describes contexts where the provider is willing to use the tongue swabs to collect samples for diagnosis of TB. |
| **SS Swab/**  **Willingness to use Supervised Self Swabbing** | Use SS Swab | With the proper training tools, I can guide my patients to swab themselves in their presence. | Describes issues related to where the provider is willing to supervise their patients to collect their own sample for TB diagnosis. |
